# Contribution of Peripheral Chemoreceptors to Exercise Intolerance in Heart Failure

**DOI:** 10.3389/fphys.2022.878363

**Published:** 2022-04-14

**Authors:** Katarzyna Kulej-Lyko, Piotr Niewinski, Stanislaw Tubek, Piotr Ponikowski

**Affiliations:** ^1^ Institute of Heart Diseases, Wroclaw Medical University, Wroclaw, Poland; ^2^ Department of Cardiology, University Clinical Hospital, Wroclaw, Poland

**Keywords:** peripheral chemoreceptors, carotid bodies, heart failure, exercise intolerance, dyspnoea, muscle fatigue

## Abstract

Peripheral chemoreceptors (PChRs), because of their strategic localization at the bifurcation of the common carotid artery and along the aortic arch, play an important protective role against hypoxia. Stimulation of PChRs evokes hyperventilation and hypertension to maintain adequate oxygenation of critical organs. A relationship between increased sensitivity of PChRs (hyperreflexia) and exercise intolerance (ExIn) in patients with heart failure (HF) has been previously reported. Moreover, some studies employing an acute blockade of PChRs (e.g., using oxygen or opioids) demonstrated improvement in exercise capacity, suggesting that hypertonicity is also involved in the development of ExIn in HF. Nonetheless, the precise mechanisms linking dysfunctional PChRs to ExIn remain unclear. From the clinical perspective, there are two main factors limiting exercise capacity in HF patients: subjective perception of dyspnoea and muscle fatigue. Both have many determinants that might be influenced by abnormal signalling from PChRs, including: exertional hyperventilation, oscillatory ventilation, ergoreceptor oversensitivity, and augmented sympathetic tone. The latter results in reduced muscle perfusion and altered muscle structure. In this review, we intend to present the milieu of abnormalities tied to malfunctioning PChRs and discuss their role in the complex relationships leading, ultimately, to ExIn.

## Introduction

Peripheral chemoreceptors (PChRs) play important roles in adapting to hypoxia, physiologically and evolutionarily. In humans, PChRs are represented mainly by carotid bodies (CBs) located close to the bifurcation of the common carotid artery and aortic bodies situated along the aortic arch (Paton et al., 2013). A decrease in arterial blood oxygenation activates CBs and then, *via* a reflex arc involving brain stem nuclei, leads to hyperventilation (Ponikowski and Banasiak, 2001). Similarly, stimulation of PChRs elicits sympathetic excitation and, in turn, increases peripheral resistance (Despas et al., 2009; Despas et al., 2012). Moreover, direct stimulation of aortic bodies and activation of Hering-Breuer reflex (originating from pulmonary stretch receptors) secondary to hyperventilation result in tachycardia (Niewinski et al., 2014; Tubek et al., 2016; Paleczny et al., 2019). Undoubtfully, these reactions are protective in acute settings (e.g., high altitude). On the other hand, elevated tonic activity and exaggerated (concerning the physiological need) acute responses from PChRs may be potentially harmful. This has repeatedly been shown in selected patients with heart failure (HF)—possibly due to enhanced adrenergic tone (Ponikowski et al., 2001a; Floras and Ponikowski, 2015).

Augmented peripheral chemosensitivity characterizes approximately 40% of patients with heart failure with reduced ejection fraction (Chua et al., 1997a; Niewinski et al., 2013). There is a clear relationship between hyperreflexia of PChRs (i.e. increased peripheral chemosensitivity) and exercise intolerance (ExIn) expressed as: 1) higher New York Heart Association (NYHA) functional class or 2) worse cardiopulmonary exercise test indices (Chua et al., 1996a; Chua et al., 1997a). Hyperreflexia of PChRs has been repeatedly linked to the worse clinical profile of HF patients, including a greater degree of neurohormonal derangement (e.g., higher level of NTpro-BNP) (Giannoni et al., 2008). However, in the experimental study employing acute inhibition of PChRs (Chua et al., 1996; Chua et al., 1997) not only oversensitivity chemoreflex but also tonic activity of PChRs have been targeted. In those studies, PChR were blocked with the use of oxygen or opioids, which resulted in improved exercise capacity of HF subjects. Whether such benefit was related to a decrease in acute reflex response to the metabolites of exercising muscles (e.g., lactate) (Torres-Torrelo et al., 2021) or to the dimished tonic activity of PChRs remains unknown.

The limitation of exertional capacity in the HF population emerges from the dyspnoea sensation and/or muscle fatigue. These are the two most common complaints reported at the end of the symptom-limited exercise test by HF patients (Clark et al., 1995). Both can be influenced by a variety of factors related primarily to low cardiac output and secondarily to: pulmonary abnormalities (Wasserman et al., 1997; Schmid et al., 2008), autonomic imbalance (Niewinski et al., 2017), impaired peripheral perfusion (Piepoli et al., 1996), and altered muscle function and structure (Wilson et al., 1993).

## Determinants of Exertional Dyspnoea in Heart Failure

### Excessive Hyperventilation During Exercise

Historical studies from the 1960s and 1970s reported a reduction of dyspnoea sensation in patients with severe bronchial asthma and chronic obstructive pulmonary disease following unilateral or bilateral CB resection (Nakayama, 1962; Winter 1973; Stulbarg and Winn, 1989). This supports a relationship between the PChRs’ function and the perception of breathing difficulty. Hyperventilation (expressed as an increase in tidal volume and/or respiratory rate) as the primary response to the activation of PChRs may be subjectively identified as shortness of breath (Chua et al., 1997a; Wasserman et al., 1997; Motiejunaite et al., 2021). Inhibition of the tonic PChRs activity (with oxygen, dopamine or dihydrocodeine) (Chua et al., 1996a; Chua et al., 1997b; van de Borne et al., 1998) or CB denervation (Niewinski et al., 2017) decreased the regression slope relating ventilation to carbon dioxide output (VE/VCO_2_ slope) in HF patients subjected to cardiopulmonary exercise test indicating diminished ventilatory effort for a given carbon dioxide production. This might translate into a reduced sensation of dyspnoea and thus explain the benefits seen in the former studies on CB resection (Nakayama, 1962; Winter 1973; Stulbarg and Winn, 1989).

A growing body of evidence points to the connection between the functionalities of central and peripheral chemoreceptors. A significant reduction of central chemosensitivity occurring acutely after bilateral CB resection is a piece of obvious evidence for the hyperadditive character of this interaction (Del Rio et al., 2013; Marcus et al., 2014). Thus, in HF, hypertonicity of PChRs enhances exertional hyperventilation not only directly but also indirectly through the augmentation of central respiratory drive.

Due to relatively stable arterial partial pressures of oxygen and carbon dioxide during incremental exercise (Sun et al., 2001; Forster et al., 2012), acute activation of PChRs is debatable in this context. On the other hand, it could be hypothesized that the rising on-exercise concentration of other (than oxygen and carbon dioxide) known CBs stimulants, such as lactate (Torres-Torrelo et al., 2021), potassium (McLoughlin et al., 1995), adenosine (McQueen and Ribeiro, 1981), and catecholamines (Lahiri et al., 1981), could contribute to excessive ventilation due to hyperreflexia of PChRs in HF. According to that notion, not only “tonic” but also “acute” reactivity of CBs would be involved in ExIn in HF patients (Scott et al., 2003). This is supported by the fact that the increase in lactate production during exercise is significantly faster in HF subjects than in healthy controls. Indeed, Scott et. (Scott et al., 2003) demonstrated that local lactate concentrations in the exercising muscles of HF patients were significantly higher than in subjects with normal left ventricular function (2.55 ± 0.2 *vs.* 1.78 ± 0.2 mmol/L).

Restriction of the inspiratory effort (“unsatisfied inspiration”) may be perceived as breathlessness limiting exercise capacity (O’Donnell et al., 1999; O’Donnell and Laveneziana, 2006). One of its main reasons is the phenomenon called dynamic lung hyperinflation (DLH), which has been described in HF patients (O’Donnell et al., 1999). DLH is characterized by a progressive rise in end-expiratory lung volume with concomitant fall in dynamic inspiratory capacity relative to the degree of air trapping. The dynamic inspiratory capacity is continuously diminished due to increasing elastic forces affecting respiratory muscles when tidal volumes are operating closer to total lung capacity.

O`Donnell et al. elegantly documented that at a peak work rate of only 41% of predicted value, end-expiratory lung volume was equal to 92% of total lung capacity in a group of stable patients with congestive HF (O’Donnell et al., 1999). DLH emerges from the expiratory flow limitation caused by several factors connected with HF state: mucosal oedema (Duguet et al., 2000), hyperresponsiveness of bronchi (Cabanes et al., 1989), and age-related airways abnormalities (Light, 1983).

Kawachi et al. show that DLH can be experimentally produced by hyperventilation (Kawachi and Fujimoto, 2020). Because hyperventilation during exercise is closely linked to PChRs’ overactivity (Chua et al., 1996a), one could expect that such patients are also prone to the development of DLH. Moreover, patients with the augmented activity of PChRs present with increased sympathetic drive (Floras and Ponikowski, 2015), which generates water and sodium retention (Martin and Schrier, 1997; Schrier, 2006). Thus, it may be speculated that interstitial oedema and inflammation within the airways produced by sympathetically-mediated [including mobilization of the venous reservoir *via* venoconstriction (Bruno et al., 2012)] increase in fluid volume (Miller, 2016) -could participate in the development of the obstructive pattern and consequently further predispose to DLH. However, such a notion would need to be confirmed in experimental studies.

Apart from the phenomena described above resulting in airway obstruction, the role of reduced lung compliance should also be emphasized (Faggiano, 1994). The restrictive pattern in HF patients may be secondary to heart enlargement, pleural effusion, and pulmonary vascular congestion with ensuing interstitial and alveolar edema (Apostolo et al., 2012). The latter results in an impairment of alveolar-capillary gas diffusion (DLCO), reflecting poor gas exchange efficiency (Agostoni et al., 2006).

### Metaboreflex Oversensitivity

Overactivity of the muscle ergoreceptors plays an important role in ExIn in HF patients (Piepoli et al., 1996; Piepoli et al., 1999; Ponikowski et al., 2001b). The ergoreceptors conduct neural traffic from the exercising muscles to the ventrolateral medulla and lateral reticular nucleus through the lateral spinothalamic tract of the spinal cord (Nauli et al., 2001). The stimulation of the ventrolateral medulla in an animal model causes a rise in arterial blood pressure, heart rate, and minute ventilation (Bauer et al., 1990). Thus, ergoreceptors are responsible for appropriate ventilation together with adequate blood supply to the working muscles (Perez-Gonzalez, 1981), which ought to be adjusted according to the local demands (Piepoli et al., 1995). Due to many biochemical and structural abnormalities in the skeletal muscles (presented below), ergoreflexes in HF patients are exaggerated, provoking an increase in ventilatory drive, overt peripheral vasoconstriction, and sympathetic excitation.

Ergoreceptors can be divided into metaboreceptors (activated by metabolites of contracting muscles) (Sinoway et al., 1993) and mechanoreceptors (sensitive to mechanical contraction) (Kaufman et al., 1983). As shown by Scott et al., the role of metaboreceptors in the development of ExIn in HF is more evident than the contribution of mechanoreceptors (Scott et al., 2000).

Interestingly, the functionality of metaboreceptors and PChRs is interrelated. Edgell et al. (Edgell and Stickland, 2014) demonstrated that the concurrent activation of metaboreceptors and CBs under hypoxic conditions leads to the augmentation of both ventilation and muscle sympathetic nerve activity (MSNA), which was not higher than the sum of each response separately. It indicates that chemoreflex activation does not increase the sensitivity of metaboreflex and *vice versa*. On the other hand, inhibition of the CBs with hyperoxia diminished sympathetic response (measured with MSNA) when concurrent metaboreflex activation was applied but did not change MSNA when metaboreflex co-activation was absent. This outcome may be explained by the change in central integration of carotid chemoreceptor feedback with metaboreflex activation. This is consistent with the notion of CBs excitation during exercise in the absence of CBs stimuli.

Contrasting results have been provided by Wan et al. (Wan et al., 2020), who presented a case for hyper-additive interaction between metabo- and chemoreflexes under normocapnic hypoxic conditions during exercise. Interestingly, a hypo-additive interaction was reported for leg blood flow and vascular conductance. Consequently, it could be hypothesized that aroused metaboreceptors may contribute to ExIn in HF due to tonic activation and possibly by augmentation of acute reflex response from PChRs. Regardless of somehow discordant results, it should be emphasized that both cited studies were carried out in healthy participants. Therefore, how these might interact in the HF population is unknown.

### Exertional Oscillatory Ventilation

Exertional oscillatory ventilation (EOV), according to the American Heart Association, is defined as an oscillatory ventilatory pattern lasting for at least 60% of the exercise duration at amplitude ≥15% of the average resting minute ventilation (Balady et al., 2010). Schmid et al. (Schmid et al., 2008) demonstrated that EOV was related to worse exercise capacity in HF. Patients with heart failure with reduced ejection fraction and EOV were characterized by poor ventilatory efficiency on exertion (higher VE/VCO_2_ slope: 38.0 ± 8.3 *vs*. 32.8 ± 6.3) and lower workload at peak exercise (ΔWatts = 5.8 ± 23.0) (Schmid et al., 2008). The potential mechanisms by which EOV attenuates exercise tolerance in HF comprise oscillatory changes of dead space ventilation and unequal lung and muscle perfusion. These disturbances generate a mismatch between ventilation and perfusion and lead to greater respiratory muscles work and higher oxygen consumption (Yajima et al., 1994; Schmid et al., 2008).

The pathophysiology of EOV and periodic breathing in HF is congruous and related to the disturbances within the “control loop” system, which regulates ventilation proportionally to the metabolic demand. These disturbances include increased controller gain (Ponikowski et al., 2001a), increased plant gain (Agostoni et al., 2002), and prolonged loop delay (Leite et al., 2003). Enhanced plant gain emerges from greater carbon dioxide damping due to diminished lung volume (Agostoni et al., 2002). Increased loop delay results from the low cardiac output- hallmark of HF (Yajima et al., 1994). Finally, augmented controller gain results from oversensitive central and peripheral chemoreceptors (Chua et al., 1996b). PChRs, as elegantly presented by Dempsey et al. (Dempsey et al., 2010; Dempsey, 2012; Dempsey et al., 2012), are essential for producing apneas following transient ventilatory overshoot and thus for periodic breathing initiation (Nakayama et al., 2003). Their hyperadditive interplay with central chemoreceptive areas in the brainstem (sensitive to carbon dioxide fluctuations) further perpetuates the oscillatory ventilation pattern.

Apart from the factors mentioned above, an augmented sensitivity of ergoreceptors (to metabolic changes occurring locally in exercising muscles), may play an additional role in generating EOV in the HF population (Dhakal and Lewis, 2016). By exaggerating the ventilatory response to exertion, sensitized metaboreceptors promote hypocapnia which, in turn, may initiate the periodic pattern of respiration in individuals characterized by abnormal components of the “control loop” (Ponikowski et al., 2001b; Scott et al., 2002).

## Determinants of Muscle Fatigue in HF

### Limited Muscle Perfusion

According to the Fick principle, oxygen consumption depends on cardiac output (CO) and peripheral oxygen utilization (VO_2_ = CO · ΔAVO_2_ [arteriovenous oxygen difference]). Consequently, individuals characterized by higher CO present with greater peakVO_2_ on exercise. Interestingly, an acute increase in CO (e.g., with catecholamines) does not influence peakVO_2_ and exercise capacity (Maskin et al., 1983). This so-called “hemodynamic paradox” can be explained only by the concurrent decline in oxygen consumption in the periphery. This brings the notion of dysfunctional peripheral tissues as the major limiting factor of exercise tolerance in HF. The oxygen consumption decreases peripherally because of: 1) restricted blood perfusion in the skeletal muscles; and 2) functional and structural abnormalities within the skeletal muscles. In healthy subjects, metabolic changes occurring in exercising muscle lead to dilatation of the local vasculature. In HF, this mechanism is limited, which could be seen as a protective mechanism aiming to preserve a minimal degree of perfusion levels for the brain, heart, and respiratory muscles (Poole et al., 2012). It is likely mediated by overactivation of the sympathetic system (a typical feature of advanced HF) (Joyner et al., 1992) combined with diminished capacity for metabolic vasodilatation (due to oxidative stress and endothelial dysfunction) (Landmesser et al., 2002; Sharma and Davidoff, 2002) leading to elevated peripheral vascular resistance (PVR). The link between PVR and tonic activation of PChRs in HF was evaluated by Tubek et al. (Tubek et al., 2021). Under hyperoxic conditions (transient administration of 100% O_2_ to inhibit PChRs), PVR decreased in the HF group (1239 ± 380 dyn s cm^−5^ *vs*. 1174 ± 299 dyn s cm^−5^, *p* < 0.05), whereas in controls there was no significant change (1180 ± 317 dyn s cm^−5^ *vs*. 1242 ± 332 dyn s cm^−5^, *p* = NS) reflecting the influence of tonic activation of PChRs over PVR in HF patients but not in healthy controls. An additional piece of evidence linking PChRs and PVR comes from the study by Niewinski et al. (Niewinski et al., 2017), in which CB resection decreased sympathetic tone measured directly with microneurography. While numerically, the decline in muscle sympathetic nerve activity (MSNA) was modest (10 bursts/100 beats at 2 months following surgery); it should be noted that it equated to ∼45% of the excess sympathetic activity related to the heart failure state (when compared with healthy volunteers of similar age) (Hart et al., 2009). Furthermore, there are some premises indicating that PChRs contribute to restriction in peripheral (not muscle) blood flow. Marcus et al. (Marcus et al., 2015) demonstrated that in rabbits with congestive heart failure, renal blood flow decreased under hypoxic conditions. This response was abolished after CB resection, confirming the maladaptive role of PChRs hyperreflexia in adequate tissue perfusion.

### Intrinsic Muscle Abnormalities

It has been suggested that intrinsic muscle dysfunction might constitute a better determinant of ExIn in the HF population than limited muscle perfusion (Wilson et al., 1993). Intrinsic muscle dysfunction ensues from structural (Wilson et al., 1993) and functional (enzymatic) alterations (Drexler et al., 1992). Among various structural changes observed in HF, a decline in the proportion of energy-efficient slow-twitch fibres (type 1) to fast-twitch fibres (type IIb; relying mostly on glycolytic metabolism) has been commonly reported (Sullivan et al., 1990).

Enzymatic changes in skeletal muscles are characterized by reduced activity of enzymes involved in aerobic metabolism without significant changes in enzymes participating in the glycolytic pathway (Sullivan et al., 1991). The function of enzymes contributing to aerobic processes is dependent on iron supply (Dziegala et al., 2018). Thus, iron deficiency evokes disturbances in the function of mitochondria in myocytes (Cartier et al., 1986), reduction of myoglobin concentration (Hagler et al., 1981), and elevation of lactate production due to impaired mitochondrial oxidative phosphorylation (Finch et al., 1979). Moreover, iron deficiency augments lipid peroxidation, which contributes to myocyte damage (Knutson et al., 2000).

Therefore, iron deficiency/anaemia (which is a common comorbidity in HF) (von Haehling et al., 2017) deteriorates skeletal muscles’ function directly (as explained above), but also indirectly–through PChRs, which become tonically activated possibly due to reduced oxygen-carrying capabilities of blood cells. In support of that notion, Franchitto et al. (Franchitto et al., 2010) demonstrated that patients with HF and anaemia are characterized by augmented baseline MSNA when compared to those with HF alone (56.0 ± 3.2 vs. 45.5 ± 3.1 bursts per min; *p* < 0.02). Furthermore, inhibition of PChR by breathing 100% oxygen for 15 min attenuated MSNA in HF patients with anaemia (from 56.0 ± 3.4 to 50.9 ± 3.2 bursts per min; *p* < 0.002) but did not alter MSNA in patients without anaemia.

An excessive sympathetic tone might translate into intrinsic muscle dysfunction by restraining muscular blood flow, increasing inflammatory cytokines production, and deterioration of energy metabolism (Nilsson Jr. et al., 2008). Activation of the *β*-adrenergic system increases glycogenolysis and lactate production in contracting muscle and enhances the uptake of oxygen and glucose (Richter et al., 1982). These effects tilt the balance between glycogenolysis and gluconeogenesis towards the unfavourable catabolic state (Richter et al., 1982). Furthermore, sympathetic overactivation promotes a surge of inflammatory cytokines–among them: tumour necrosis factor-α, which has known proapoptotic properties (Dalla Libera et al., 2001) and interleukin-6, whose level is inversely related to muscle fibre diameter (Larsen et al., 2002).

### Deranged Central Hemodynamics

Central hemodynamics, expressed as a cardiac index or pulmonary artery wedge pressure, do not correlate with exercise tolerance characterized by peakVO_2_ in patients with advanced HF (Wilson et al., 1995). On the other hand, there is no doubt that low cardiac output by itself is the primary reason for most of the disturbances (mentioned in the above paragraphs) that finally culminate in the development of ExIn. Interestingly, studies with biventricular cardiac resynchronization therapy, where an acute augmentation of cardiac output improves exercise tolerance, support this concept (Laveneziana et al., 2009; Schlosshan et al., 2009). This beneficial effect of cardiac resynchronization is multifactorial and likely related to several pathways of action: ergoreceptors modulation (Jaussaud et al., 2012), improvement in respiratory muscles’ function (improved dynamic inspiratory capacity) (Papazachou et al., 2007), and attenuation of the sympathetic drive (Hamdan et al., 2002).

To date, we are not aware of any published data documenting that cardiac resynchronization therapy or other intervention acutely enhancing cardiac output influences the activity of PChRs. On the other hand, as showed in the rabbit model by Ding et al. (Ding et al., 2011), experimental reduction of CB perfusion (using carotid artery occluders mimicking diminished blood flow as seen in HF state) augments peripheral chemosensitivity. Similarly, Del Rio et al. (Del Rio et al., 2017) demonstrated that in rats with ischaemic HF, augmentation of chemoreflex was related to reduced cardiac output. Interestingly, animals with low cardiac output exhibited a trend towards reduction of Krüppel-like Factor 2 (KLF2) expression in CBs (Del Rio et al., 2017). The downregulation of KLF2 (a shear stress-sensitive transcription factor) leads to oversensitivity of PChRs, increase in renal sympathetic nerve activity, development of oscillatory breathing, and propensity for arrhythmias in rabbits with congestive HF (Marcus et al., 2018). Interestingly, increasing KLF2 expression with simvastatin treatment in rodent model limited the augmentation of peripheral chemosensitivity and improved respiratory variability, periodic breathing and arrhythmia index following coronary ligation (Haack et al., 2014).

### The Impact of Exercise Training on Peripheral Chemoreflex Function

Some premises suggest that exercise training (ExT) may normalize the oversensitivity of peripheral chemoreflexes (Schultz et al., 2015). Calegari et al. (Calegari et al., 2016) presented that regular treadmill for 8 weeks (60 min/day, 5 days/week) improved baroreflex sensitivity and the attenuated acute pressor response elicited by potassium cyanide in HF rats. In another study, Li et al. (Li et al., 2008) investigated the impact of ExT on peripheral chemoreflex in rabbits with congestive HF. They found that 4–5 weeks of treadmill training (30–40 min/day, 6 days/week) decreased tonic single-fiber discharge within the CB nerve and reduced the acute response to hypoxia. Furthermore, ExT attenuated elevated angiotensin II levels and increased nitric oxide concentration. Downing and Balady (Downing and Balady, 2011) suggested that restoration of sympatho-vagal balance contributes to improved exercise tolerance seen after regular ExT in HF patients. This beneficial change might be mediated by increased blood flow through CBs occurring during repetitive exercise, which desensitizes PChRs (through upregulation of KLF2) and, in turn, decreases adrenergic tone (Marcus et al., 2018).

## Summary

**FIGURE 1:**
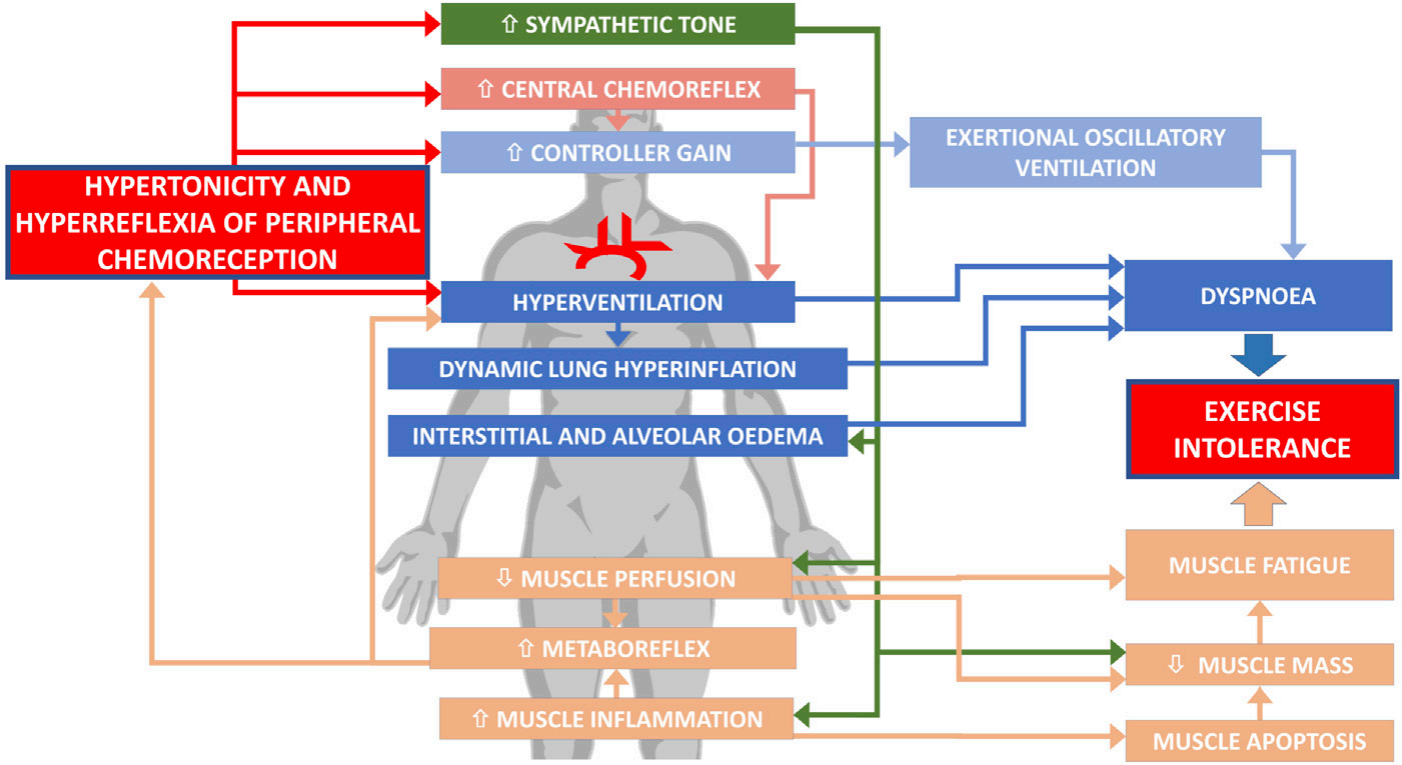
Involvement of abnormal peripheral chemoreception in exercise intolerance in heart failure.

The pathophysiology of ExIn in HF is neither simple nor intuitive. Numerous factors evoking ExIn exceed beyond low cardiac output and are closely interrelated. The impaired function of PChRs–both an augmented tonic activity (hypertonicity) and increased acute sensitivity (hyperreflexia)—presents as an important link in the complex pathophysiology of poor exercise capacity in HF (Figure 1). As discussed above, the detrimental role of PChRs is related to both dyspnea sensation and muscle fatigue, with sympathetic overactivation and hyperventilation as the major mediators leading to ExIn.

Therefore, PChRs seem to be an attractive goal for novel therapies aiming to improve exercise tolerance in the HF population. Importantly, such interventions due to the protective role of PChRs against hypoxemia ought to be performed with great caution (Niewinski et al., 2021). Bilateral CB resection might result in profound blood oxygen desaturation and marked variability in saturation levels even during mild hypoxia (Niewinski et al., 2021). One way forward might be to use a pharmacological modulation (e.g., using specific P2X3 inhibitors) instead of an irreversible surgical approach (Pijacka et al., 2016). Recently proposed denervation of the sympathetic ganglioglomerular nerve, which is involved in the tonic activation and sensitization of CB, would also maintain the physiological function of PChRs, thereby minimizing hypoxia-related side effects (Niewinski et al., 2021). Those methods of selective modulation of PChRs, while attractive from a conceptual point of view, have not yet been transferred into human clinical trials. Only by performing randomized and placebo (or sham) controlled studies one could unravel the true role of PChRs in ExIn.
